# Tracheoesophageal Fistula Following Chemoradiation and Esophagectomy in Esophageal Adenocarcinoma: A Fatal Complication

**DOI:** 10.7759/cureus.87394

**Published:** 2025-07-06

**Authors:** Mujtaba Moazzam, Usman Bin Hameed, Saman Amjad, Fady Banno, Aleena Salman

**Affiliations:** 1 Internal Medicine, Corewell Health William Beaumont University Hospital, Royal Oak, USA; 2 Gastroenterology, Corewell Health William Beaumont University Hospital, Royal Oak, USA; 3 Medicine, Shifa College of Medicine, Shifa Tameer-e-Millat University, Islamabad, PAK

**Keywords:** chemoradiation complications, esophageal adenocarcinoma, esophagectomy complications, immunotherapy-related adverse effects, tracheoesophageal fistula, tracheostomy dislodgement, tracheostomy in tef

## Abstract

Tracheoesophageal fistula (TEF) is a rare but devastating complication that can arise in patients with esophageal cancer following multimodal therapy. We present the case of a 45-year-old male with stage III esophageal adenocarcinoma treated with neoadjuvant chemoradiation, followed by esophagectomy and immunotherapy. He subsequently developed a large TEF, resulting in recurrent aspiration, bleeding, and ultimately death despite comprehensive supportive measures. TEFs are associated with significant morbidity and mortality, and current therapeutic strategies remain limited. However, promising advances in endoscopic modalities and reconstructive surgery are emerging. Management necessitates a multidisciplinary, individualized approach, although standardized guidelines are currently lacking. Our case underscores the need for vigilance in patients at risk for TEF and the critical importance of early diagnosis and tailored intervention to prevent life-threatening complications.

## Introduction

Tracheoesophageal fistulas (TEFs) are abnormal connections between the esophagus and tracheobronchial tree, usually acquired in adults due to malignancy, chronic inflammation, or iatrogenic injury. TEF is a rare yet severe complication, seen in approximately 5-15% of individuals with esophageal cancer and in 1-5% of those with tracheobronchial malignancies [1]. TEFs may develop from direct tumor invasion or as a result of therapies. Radiation and chemotherapy can induce tumor necrosis and tissue breakdown, leading to fistulization [1]. Post-surgical fistulas are uncommon, with an incidence of 0.3-1% after esophagectomy [2]. However, the risk is significantly elevated in patients who receive radiotherapy or other adjunctive therapies. For instance, unresectable tumors invading the trachea (T4b disease) treated with definitive chemoradiation have shown esophageal fistula rates of up to 30% [3]. Although immunotherapy has improved survival in recent years, rare therapy-associated fistulas have been reported [4].

The clinical relevance of TEFs cannot be overstated: even a small TEF can lead to severe aspiration, recurrent pneumonia, sepsis, and catastrophic hemorrhage if major blood vessels are eroded. These severe complications lead to high morbidity and mortality rates, with death occurring in up to 40% of cases. Patients with malignant TEFs often have advanced disease and poor quality of life due to dysphagia, cough, and recurrent infections, posing significant management challenges [1]. Here, we present a complex TEF case after multimodal therapy, highlighting diagnostic considerations, treatment challenges, and evolving interventions.

## Case presentation

A 45-year-old male was diagnosed with stage III (T3N1M0) esophageal adenocarcinoma in September 2022. He initially received concurrent chemoradiotherapy, followed by esophagectomy with gastric pull-through in October 2022, and was maintained on nivolumab. Over the next year, he developed oligometastatic disease involving the paratracheal lymph nodes and the left adrenal gland, for which he received systemic chemotherapy (FOLFOX) and subsequently remained on pembrolizumab maintenance therapy. Follow-up imaging demonstrated progression of the right paratracheal lymph node, which was unresectable. In October 2024, he underwent radiofrequency ablation (RFA) and continued maintenance immunotherapy.

Two months later, he presented to the emergency department following an unwitnessed syncopal episode at home, preceded by a three- to five-day history of fatigue, malaise, palpitations, and melena. He also noted the development of new-onset dysphagia after undergoing RFA, which had progressively worsened over the past week. On arrival, he was hypotensive (BP 82/36 mmHg), tachycardic (HR 140 bpm), and mildly hypoxic on 2 L/min of oxygen. Laboratory studies (Table 1, Table 2) showed profound anemia (hemoglobin 5.8 g/dL), neutrophilic leukocytosis (white blood cells 29,000), normal platelet count (220,000), elevated lactic acid (3 mmol/L), normal liver enzymes, and elevated creatinine (1.8 mg/dL).

**Table 1:** Complete blood count demonstrating anemia and leukocytosis

Parameter	Admission value	Reference range (units)
White blood cells	29	3.5-10.1 bil/L
Red blood cells	2.04	4.31-5.48 tril/L
Hemoglobin	5.8	13.5-17 g/dL
Hematocrit	17.9	40.1-50.1%
Mean corpuscular volume	87.7	80-100 fL
Mean corpuscular hemoglobin	28.4	27-33 pg
Mean corpuscular hemoglobin concentration	32.4	32-36 g/dL
Red cell distribution width	15.7	12-15%
Platelet count	352	150-400 bil/L

**Table 2:** Comprehensive metabolic panel demonstrating elevated creatinine and lactate

Parameter	Admission value	Reference range (units)
Sodium level	134	135-145 mmol/L
Potassium level	3.4	3.5-5.1 mmol/L
Chloride	106	98-107 mmol/L
Bicarbonate level	18	22-29 mmol/L
Anion gap	10	6-12
Blood urea nitrogen	24	7-20 mg/dL
Creatinine	1.8	0.6-1.3 mg/dL
Estimated glomerular filtration rate by creatinine	42	≥90 mL/min/1.73 m²
Glucose level	234	70-110 mg/dL
Calcium level total	7.9	8.5-10.5 mg/dL
Phosphorus level	2.7	2.5-4.5 mg/dL
Magnesium	1.4	1.5-2.5 mg/dL
Alkaline phosphatase	50	44-147 IU/L
Albumin level	2.5	3.4-5.4 g/dL
Protein total	5.3	6.0-8.3 g/dL
Aspartate aminotransferase	15	5-40 IU/L
Alanine aminotransferase	7	7-56 IU/L
Bilirubin total	0.3	0.1-1.2 mg/dL
Lactic acid	3	0.5-2.2 mmol/L

**Figure 1:**
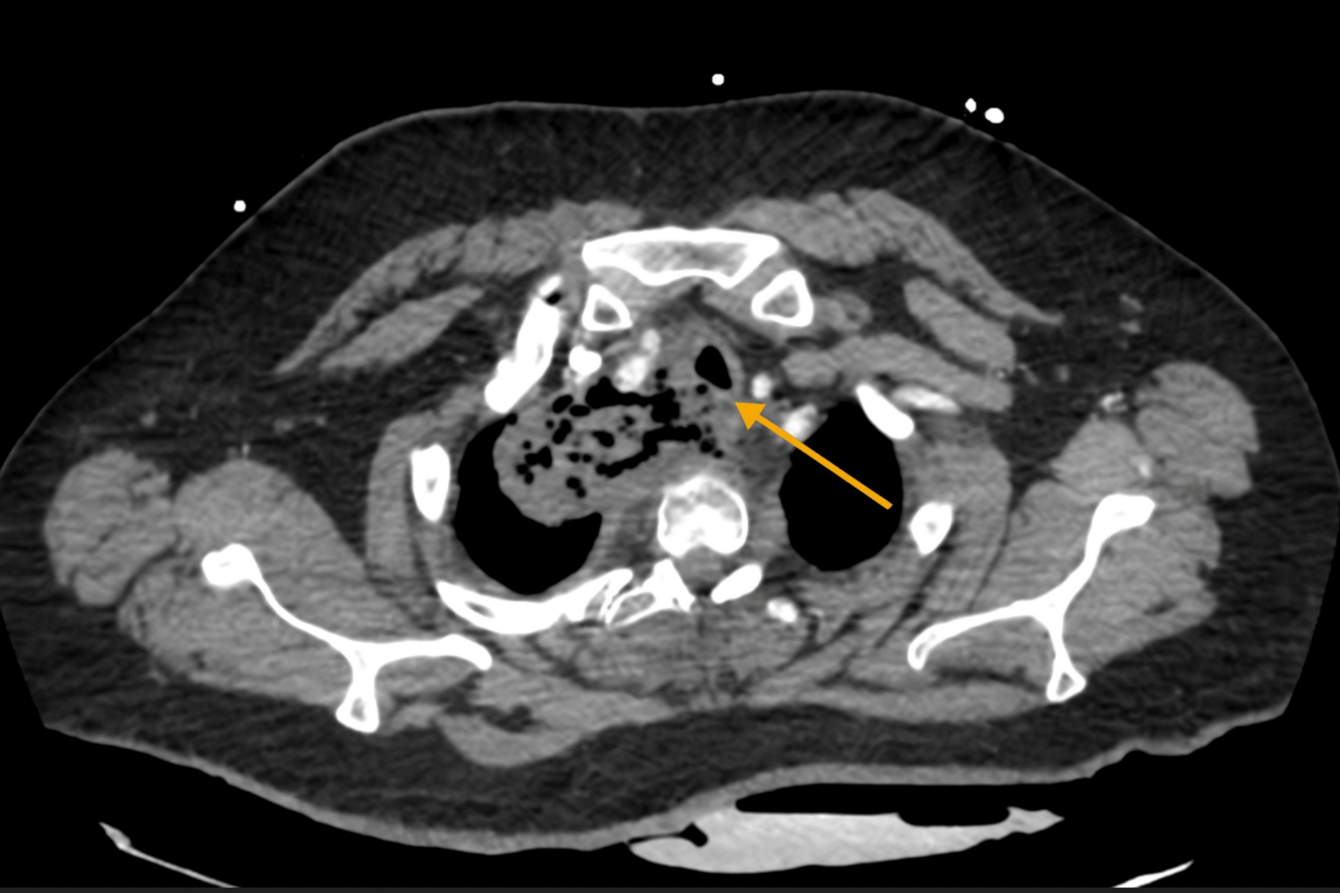
CT of the thorax showing esophageal dehiscence in the right paratracheal region, concerning for esophageal perforation

Initial management included multiple packed red blood cell transfusions, intravenous fluids, broad-spectrum antibiotics, and transfer to the ICU for monitoring. A CT thorax revealed esophageal dehiscence in the right paratracheal area with the presence of gas and food debris, raising concern for esophageal perforation (Figure 1).

**Figure 2:**
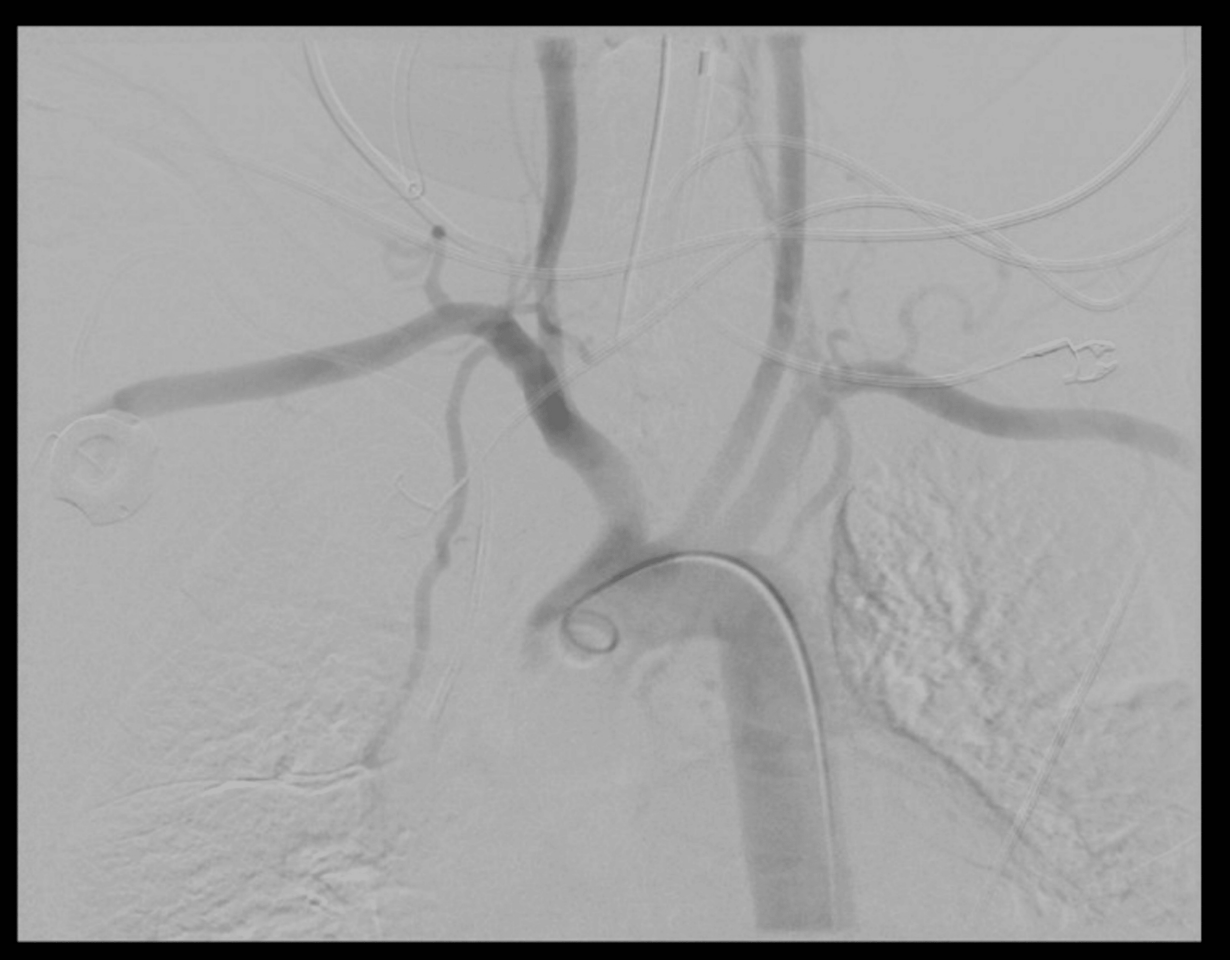
Thoracic angiogram showing no evidence of active extravasation or vascular abnormality The study was performed in the context of massive hemoptysis to evaluate potential bleeding sources; however, no active arterial bleeding was identified.

**Figure 3:**
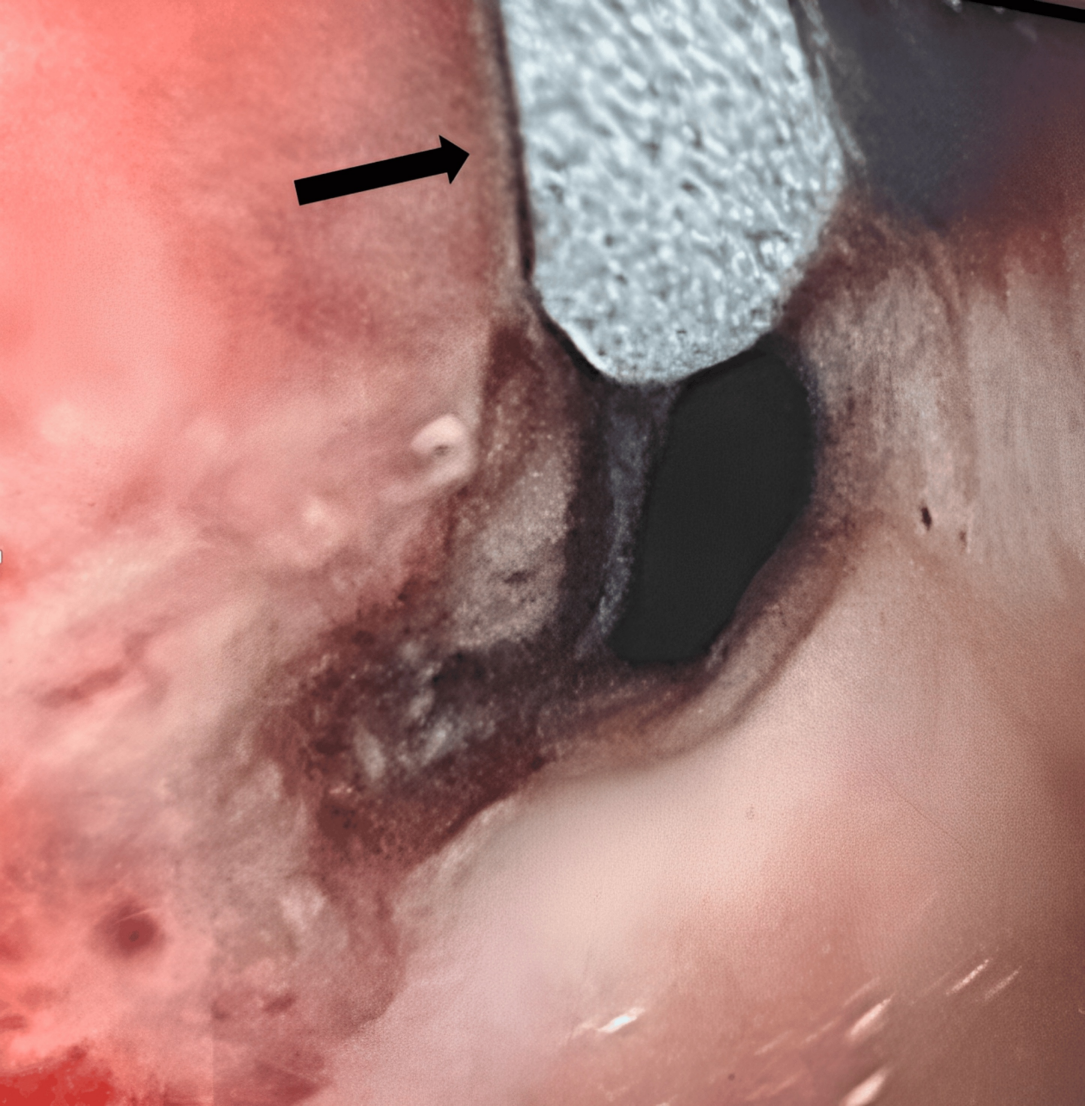
Endoscopic image showing a large tracheoesophageal fistula approximately 20 cm from the incisors, located in the mid-trachea The endotracheal tube cuff is visible protruding into the esophageal lumen through the tracheoesophageal fistula.

**Figure 4:**
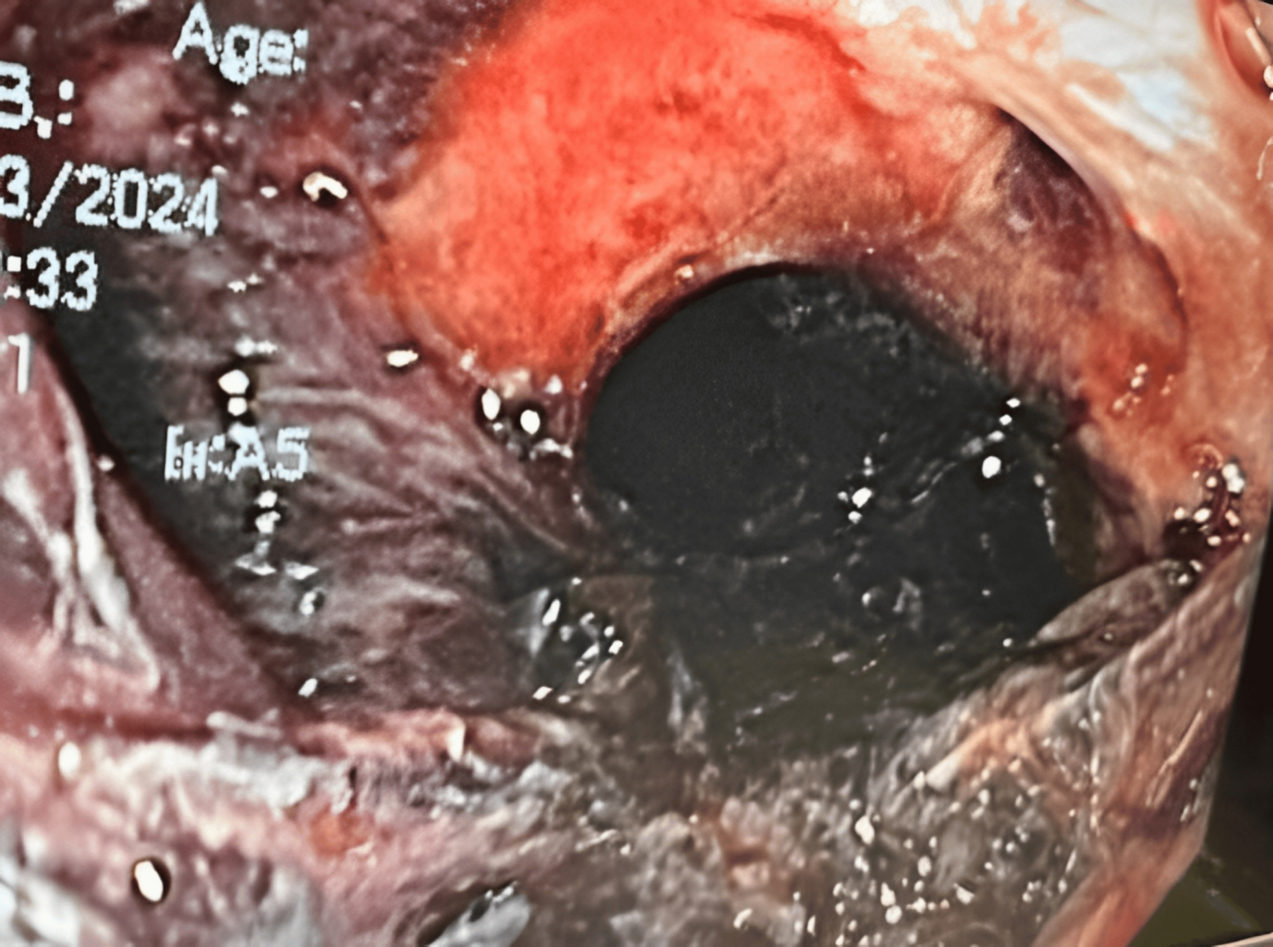
Endoscopic image demonstrating intraluminal esophageal bleeding, likely triggered by endotracheal tube cuff deflation in the setting of a tracheoesophageal fistula

**Figure 5:**
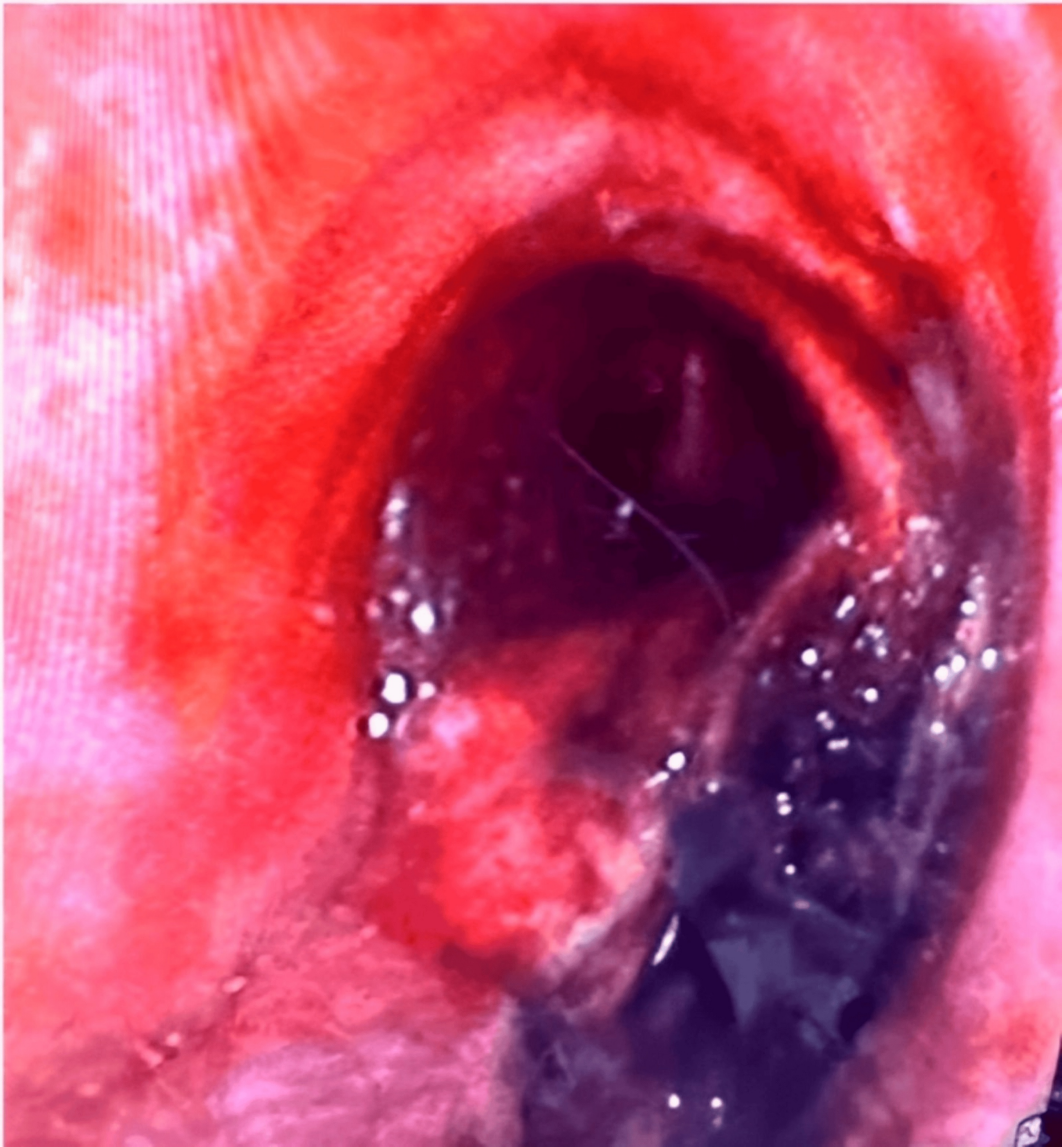
Bronchoscopy findings showing a large perforation of the right posterior tracheal wall with communication to the esophagus, located approximately 6-8 cm above the carina Upon deflation of the endotracheal tube balloon, a continuous stream of dark, bloody fluid was observed flowing into the airway.

Within 24 hours, he developed massive hematemesis and hemoptysis, requiring emergent intubation. Bedside bronchoscopy showed extensive blood in the airways without an identifiable bleeding source. Thoracic and mesenteric angiography (Figure 2) also failed to reveal an active site of hemorrhage. Subsequent EGD performed with GI and thoracic surgery confirmed a large TEF approximately 20 cm from the incisors at the mid-trachea (Figure 3, Figure 4). Repeat bronchoscopy demonstrated a large posterior tracheal wall defect with continuous bloody secretions (Figure 5).

Definitive surgical repair was not feasible due to prior radiation, surgery, and severely friable tissue, which carried a high risk of catastrophic dehiscence. Likewise, stent placement was not an option because of the risk of erosion in the irradiated tissues and because the distal end of the stent would extend into the gastric conduit, preventing an adequate seal and allowing persistent tracheal contamination.

A multidisciplinary team opted for palliative airway management: a distal tracheostomy was placed below the fistula, with strict nil per os (NPO) status and enteral feeding via jejunostomy to minimize further injury and allow for possible spontaneous closure. Unfortunately, the tracheostomy tube dislodged into the fistula, causing acute hypoxia and bradycardia, necessitating emergent removal and endotracheal tube reinsertion. He continued to experience recurrent bleeding and hypoxic episodes, requiring multiple bronchoscopic interventions. Ultimately, a catastrophic hemorrhage led to profound hypoxia and cardiac arrest. Given his poor prognosis and lack of viable curative options, his family elected for comfort measures, and the patient died shortly thereafter.

## Discussion

Acquired TEFs in esophageal cancer patients, while rare, are well documented. After esophagectomy, TEFs occur in less than 1% of cases, with Wang et al. reporting an incidence as low as 0.4% in over 6,300 cases [2]. Conversely, tumors treated with definitive chemoradiation pose a much higher risk, with reported incidence rates of 5-15% but can reach up to 30%, as reported by Cheng et al. [3].

Multiple patient and treatment factors contribute to TEF risk. Large, ulcerative malignancies that directly invade the tracheobronchial tree markedly increase the risk of fistula formation [5]. High-dose mediastinal radiation can injure healthy tissues and promote tumor necrosis, resulting in structural weakness of the esophageal and tracheal walls; this risk is even higher with repeat radiation exposure [1,5]. Although less common, immune checkpoint inhibitors like nivolumab and pembrolizumab have been associated with TEFs in patients with prior therapies [6], and clinical trials have documented a small but notable incidence of esophageal fistulas among patients receiving nivolumab [4]. Surgical factors are also significant: TEFs following esophagectomy may result from technical challenges or impaired healing. Anastomotic leaks or necrosis of the gastric conduit can lead to mediastinal infection or abscess formation, which can erode into the airway [1].

Our patient had multiple contributing risk factors: esophageal adenocarcinoma treated with chemoradiation, major surgery, and subsequent immunotherapy, all of which likely compromised tissue integrity. His postoperative radiation changes and immune response may have hindered healing and facilitated erosion once a microperforation developed. The fistula formed near the tracheal carina, close to the esophagogastric anastomosis, suggesting that a small anastomotic leak or ulcer, possibly due to tumor recurrence or immunotherapy-induced esophagitis, progressively enlarged into a TEF. Additionally, prior RFA of the right paratracheal lymph node may have further contributed to local tissue breakdown and fistula formation.

The clinical presentation of a TEF depends on its size and location, but typically includes persistent coughing or choking while swallowing. Aspiration pneumonia is common due to leakage of oral or gastric contents into the airway, leading to cough, fever, and dyspnea [1,2]. Larger fistulas can allow gastric acid into the airway, causing bronchospasm or chemical pneumonitis. If the fistula erodes into major blood vessels, patients may develop massive hemoptysis or hematemesis, as occurred in our patient, and it is often a precursor to catastrophic hemorrhage.

Prompt diagnosis of a TEF is essential, as early intervention can prevent life-threatening aspiration and deterioration. A high index of suspicion is warranted in any post-esophagectomy patient with new respiratory symptoms or any esophageal cancer patient developing unexplained pulmonary issues. Endoscopy is the diagnostic tool of choice: combined bronchoscopy and endoscopy allow direct visualization from both sides [1]. In our case, bronchoscopy helped localize the area (noting pooled secretions in the right main bronchus), and endoscopy identified the fistula opening about 20 cm from the incisors. Imaging can also provide indirect evidence and delineate any associated abscess or pneumonitis, as in our case, where it showed esophageal dehiscence.

Clinically, once a TEF develops, patients quickly enter a cycle of aspiration and infection. Most require prompt airway protection via intubation or tracheostomy and alternative nutrition through a feeding tube. Without timely intervention, mortality is high due to severe pneumonia, sepsis, or massive hemorrhage. One series reported that sepsis and respiratory failure, rather than the underlying cancer, were the leading causes of death in patients with malignant TEF [1].

TEF management is complex and must be tailored to the patient’s condition, fistula size and location, and underlying cancer status. The main goals are to separate the respiratory and digestive tracts to prevent airway contamination and to maintain adequate nutrition [1,7]. A multidisciplinary team is essential. Treatment strategies may be conservative, endoscopic, surgical, or a combination [7].

Initial management focuses on stabilizing the patient: securing the airway with endotracheal intubation or tracheostomy positioned distal to the fistula, strict NPO status, and antibiotics [1,7]. Nutritional support via gastrostomy or jejunostomy should be established [1]. In selected cases, especially small postoperative fistulas, conservative measures may allow spontaneous closure [2]. In our patient, conservative measures such as tracheostomy, tube feeding, and antibiotics were initiated but were not successful, and our patient decompensated rapidly.

Over half of malignant TEFs eventually require intervention [2]. Endoscopy plays a key role, with stent placement being the most common intervention [1,7]. It is considered first-line palliative therapy for malignant TEF, but complications like migration and erosion are common [2,8,9]. Regardless, stenting can quickly reduce aspiration and often allows patients to resume oral intake [1]. However, in our patient, prior surgery and radiation precluded safe stenting due to the risk of erosion in the irradiated tissues, and because the distal end of the stent would extend into the gastric conduit, preventing an adequate seal and allowing persistent tracheal contamination.

Definitive surgical repair remains the only potential cure but is rarely feasible in malignant cases due to poor tissue quality or patient frailty, as was seen in our patient [1,7]. Many centers first stabilize malignant TEFs with stents and consider surgery later if infection resolves and oncological status allows [7].

Overall, the prognosis of malignant TEFs remains poor: median survival after malignant TEF is typically three to six months [5]. Most deaths result from aspiration pneumonia, sepsis, or hemorrhage [1,7]. In a review by Lenz et al., fewer than 20% of patients with malignant TEF survived over a year [10].

Emerging approaches and technologies offer some hope. Endoscopic innovations like over-the-scope clips and even endoscopic vacuum-assisted closure, a technique widely used for esophageal anastomotic leaks, are being adapted for TEF management in select cases [11]. Tissue engineering with bioprosthetic plugs has shown promise [12]. Enhanced surgical flaps, such as omental or intercostal muscle, may improve outcomes in select patients [8]. Cardiac septal occluder devices, including off-label use of patent foramen ovale occluders, have also been reported as a novel palliative strategy to seal fistulas and reduce aspiration when conventional stenting is not feasible [13].

## Conclusions

This case highlights the devastating consequences of TEF formation following multimodal treatment for esophageal cancer. Despite aggressive interventions and multidisciplinary care, malignant TEFs remain a highly morbid condition with limited curative options. Our patient’s clinical course underscores the critical importance of early recognition, vigilant surveillance in high-risk individuals, and individualized management strategies. As endoscopic and surgical innovations evolve, future advancements may offer improved outcomes in selected patients. Until then, a high index of suspicion and timely intervention remain essential in mitigating the life-threatening complications of this rare but formidable entity.
